# Action planning as predictor of health protective and health risk behavior: an investigation of fruit and snack consumption

**DOI:** 10.1186/1479-5868-6-69

**Published:** 2009-10-13

**Authors:** Liesbeth van Osch, Mariëlle Beenackers, Astrid Reubsaet, Lilian Lechner, Math Candel, Hein de Vries

**Affiliations:** 1 Department of Health Education and Health Promotion, Maastricht University, Care and Public Health Research Institute (CAPHRI), PO Box 616, 6200 MD, Maastricht, the Netherlands; 2 Faculty of Psychology, Open University Netherlands, Care and Public Health Research Institute (CAPHRI), PO Box 2960, 6401 DL, Heerlen, the Netherlands; 3 Department of Methodology and Statistics, Maastricht University, Care and Public Health Research Institute (CAPHRI), PO Box 616, 6200 MD, Maastricht, the Netherlands

## Abstract

**Background:**

Large discrepancies between people's intention to eat a healthy diet and actual dietary behavior indicate that motivation is not a sufficient instigator for healthy behavior. Research efforts to decrease this 'intention - behavior gap' have centered on aspects of self-regulation, most importantly self-regulatory planning. Most studies on the impact of self-regulatory planning in health and dietary behavior focus on the promotion of health protective behaviors. This study investigates and compares the predictive value of action planning in health protective behavior and the restriction of health risk behavior.

**Methods:**

Two longitudinal observational studies were performed simultaneously, one focusing on fruit consumption (N = 572) and one on high-caloric snack consumption (N = 585) in Dutch adults. Structural equation modeling was used to investigate and compare the predictive value of action planning in both behaviors, correcting for demographics and the influence of motivational factors and past behavior. The nature of the influence of action planning was investigated by testing mediating and moderating effects.

**Results:**

Action planning was a significant predictor of fruit consumption and restricted snack consumption beyond the influence of motivational factors and past behavior. The strength of the predictive value of action planning did not differ between the two behaviors. Evidence for mediation of the intention - behavior relationship was found for both behaviors. Positive moderating effects of action planning were demonstrated for fruit consumption, indicating that individuals who report high levels of action planning are significantly more likely to translate their intentions into actual behavior.

**Conclusion:**

The results indicate that the planning of specific preparatory actions predicts the performance of healthy dietary behavior and support the application of self-regulatory planning in both health protective and health risk behaviors. Future interventions in dietary modification may turn these findings to advantage by incorporating one common planning protocol to increase the likelihood that good intentions are translated into healthy dietary behavior.

## Background

Achieving and maintaining a healthy diet is all about consuming adequate amounts of wholesome nutrition and restricting the consumption of unhealthy, high-caloric foods. If only motivation would be enough for people to eat healthily, we would not be faced with the alarming figures on overweight and obesity. In the Netherlands, high levels of motivation (e.g., 85% of non-obese adults have a positive intention to prevent weight gain, [1]) sharply contrast with the approximate doubling of obesity prevalence over the last twenty years. Moreover, although approximately 60 to 80% of the Dutch adult population indicate that they intend to eat more fruit and less fat [2-4], only 30% of Dutch adults consume sufficient amounts of fruit and approximately one in ten individuals comply with recommendations on saturated fat intake [5].

This discrepancy between motivation and actual health behavior has been subject to profound scrutiny in the past decade. In a review of health behaviors [6], it was demonstrated that only 53% of individuals with positive intentions to engage in a health behavior translate their intentions into actual behavior. Furthermore, intentions have generally been found to account for only 20% to 40% of variance in behavior and behavior change [7-9]. These findings conflict with most traditional social-cognitive theories, such as the Theory of Planned Behavior [10], Social Cognitive Theory [11], and Protection Motivation Theory [12], that consider intention as the most proximal and powerful predictor of health behavior and indicate that other, postmotivational processes are essential in the translation of intentions into behavior. Research efforts to narrow the intention - behavior gap have centered on aspects of self-regulation [13,14] and have resulted in the proposition of planning cognitions as an important volitional factor affecting behavior and behavior change. Notions of these efforts are reflected in recent social cognition models such as the Health Action Process Approach [15] and the I-Change Model [16,17], that acknowledge and demonstrate he importance of self-regulatory planning as an important factor in the translation of intentions into behavior.

*Action planning *encompasses setting goals and planning specific actions in the striving for these goals. A substantial amount of studies that have recently been performed with regard to the impact of action planning have centered on the concept of *implementation intentions *[18,19] and related planning concepts in which plans are formulated that specify when, where, and how one intends to perform a specific behavior [20,21]. Several published experimental studies have demonstrated efficacy of action planning in the promotion of health behaviors, such as physical activity [e.g., [21,22], but see [23]] and healthy dietary intake [[24-26]; but see [27]; for reviews see [28,29]]. Furthermore, several correlational studies point towards action planning as a potentially important cognition in the transition of intentions to health behaviors [e.g., [30-32]]. With regard to the nature of its behavioral influence, action planning has been found to mediate as well as moderate the intention - behavior relationship [e.g., [32-35]].

However, contrary to a relatively large amount of studies that examine the influence of action planning with regard to health promoting and health protective behaviors, there is a notable lack of studies investigating its influence on health risk behaviors, i.e. behaviors that should be reduced, ceased or prevented in order to benefit health [28], such as smoking, (excessive) alcohol consumption, and the consumption of unhealthy foods. In these latter 'avoidance behaviors', the goal behavior is to suppress and avoid an unwanted response (e.g. eating an unhealthy snack), whereas so far, most literature with regard to action planning has focused on 'approach behaviors' that imply the initiation of a desired response (e.g. eating fruit). With regard to nutrition behavior, there have been only four studies published that report on the influence of planning on the restriction of unhealthy eating [24,26,35,36]. Although these studies varied in the applied forms of planning - the content of the formulated plans ranged from distraction-inhibition [35] to approach goals [24,26] and avoidance goals [36] - they tentatively indicate that action planning may be effectively applied to the restriction of health risk behaviors. The study by Verplanken and Faes, however, demonstrated that although the planning manipulation resulted in healthier dietary behavior, it did not break the negative influence of counterintentional, unhealthy habits, such as eating fatty snacks and sweets.

All but one study [35] used general dietary assessments as their main outcome measure (e.g. mean daily calorie or fat intake), which makes it difficult to unravel the origin of dietary changes and compromises the interpretation of the effects of action planning in restricting unhealthy eating; a reduction in mean caloric intake can be brought about by a decrease in unhealthy, high-caloric food intake, as well as an overall lower food intake of both unhealthy and healthy foods, or even a lower intake of healthy foods. In order to ascertain that a decrease in unhealthy food consumption is the single cause of reductions in general measures of caloric or fat intake, congruence between the content of action plans and the outcome measures is required. Therefore, consumption measures of separate food categories (e.g. healthy snacks vs. unhealthy snacks) are necessary.

Taken together, these findings and considerations establish the need for a more thorough investigation and comparison of the influence of action planning in the promotion of health protective behaviors and the restriction of health risk behaviors. Outcomes of this comparison may be particularly relevant in the area of dietary behavior change as achieving and maintaining a healthy diet implies both types of behaviors (i.e. the consumption of healthy foods should be increased, whereas the consumption of unhealthy foods should be decreased). If action planning proves to be important in bridging the intention - behavior gap of both types of behaviors, future interventions may benefit from the use of a single type of planning and one common planning protocol for dietary behavior change.

In line with this reasoning, the present manuscript describes two separate, yet simultaneously performed, longitudinal studies that investigate and compare the predictive value of action planning in health protective behavior and health risk behavior with regard to nutrition. The health protective behavior under study is the consumption of fruit; the health risk behavior pertains to the restricted consumption of high-caloric snacks. Where most previous observational studies have failed to incorporate a measure of past behavior, which is generally the most powerful predictor of future behavior, the present study adequately accounted for the influence of past behavior. This enabled the investigation of the value of action planning in the prediction of behavior as well as behavior change. We hypothesized that action planning would positively predict the performance of both health protective behavior and the restriction of the health risk behavior (hypothesis 1). Based on previous findings with regard to the nature of the influence of action planning [e.g., [30-34,37]], we furthermore hypothesized that action planning mediates as well as moderates the intention - behavior relationship (hypothesis 2). Lastly, we expected that the predictive value of action planning would be equally strong in both types of behaviors (hypothesis 3).

The action planning concept under investigation involves the planning of specific (preparatory) actions [e.g., [34,38-41]] that facilitate the performance of the ultimate goal behavior. This type of action planning is based on goal-setting theory [42,43], assuming that when people are faced with specific goals (e.g. daily consumption of fruit), they tend to formulate plans and task strategies on how the goal can be reached [42,44,45]. The development of these action plans predetermines a consecutive course of action (e.g., buying fruit, taking fruit along when you go to work, substituting snacks by fruit, etcetera) that is aimed at facilitating goal achievement.

## Methods

### Procedure

Two separate studies were performed simultaneously, one focusing on fruit consumption and the other focusing on the consumption of high-caloric snacks. Both study samples consisted of Dutch adults (> 18 years) that were all registered members of an online survey panel of a private research company. A total of 806 participants were invited by e-mail to participate in the online study on fruit consumption and 807 participants were invited to participate in the online study on snack consumption. Invitations were study-specific, i.e., it was not possible for individuals to participate in both studies. Participants were explained that confidentiality would be ensured, that the concerning study would comprise three measurements and that they would receive a small incentive (approximately € 3) after completing all three questionnaires. By activating a link in the e-mail, participants were directed to the web page where they could fill out the questionnaire.

At the baseline measurement (T1), 572 respondents (71.0%) filled out the questionnaire on fruit consumption and 585 respondents (72.5%) filled out the questionnaire on snack consumption. In the first follow-up measurement one month later (T2), 498 respondents participated in the fruit study (87.1% of baseline) and 508 respondents participated in the snack study (86.8% of baseline), whereas a total of 434 respondents in the fruit study (75.9% of baseline) and 442 respondents in the snack study (75.6% of baseline) had completed all three questionnaires at the second follow-up measurement two months after baseline (T3).

### Questionnaires

In the baseline questionnaires of both studies, relevant demographic variables, past behavior (previous fruit or snack consumption) self-efficacy and intention were measured. At T2 (one month after baseline), action planning was measured, and at T3 (two months after baseline) the outcome behavior (current fruit or snack consumption) was assessed. The target behaviors that were mentioned in all questions were 'eating a sufficient amount of fruit each day', which was previously explained to participants as 'two pieces of fruit each day', and 'eating as little high-caloric snacks as possible', i.e. restricting the consumption of snacks.

#### Demographics (T1)

Gender, age, and highest completed educational level were inquired after. Educational level was categorized into 'low' (elementary education, medium general secondary education, preparatory vocational school, or lower vocational school), 'medium' (higher general secondary education, preparatory academic education, or medium vocational school) and 'high' (higher vocational school or university level).

#### Self-efficacy (T1)

Self-efficacy expectations were measured by four items in each study and asked to what extent respondents think they will be able to perform the target behavior in various situations [e.g., [46]]. For fruit consumption, these situations pertained to 'during the week', 'during the weekend', 'when you are very busy', and 'during the winter months' (Cronbach's α = 0.91). For snack consumption, these situations pertained to 'during the weekend', 'when you are very busy', 'when you are at a party', and 'when you have a craving for snacks' (Cronbach's α = 0.81). Answering options for each item ranged from 'I will certainly not be able to' (1) to 'I will certainly be able to' (7).

#### Intention (T1)

Intention was measured by two items in each study. The first item asked to what extent respondents intended to perform the target behavior [e.g., [47]]. In the second item a time-reference was added, asking respondents to what extent they intended to perform the target behavior in the next month [e.g., [48,49]]. For both questions, answering options ranged from 'I definitely do not intend to' (1) to 'I definitely intend to' (7). Reliability of the intention measure was high in both studies (fruit: Cronbach's α = 0.93; snack: Cronbach's α = 0.96).

#### Action planning (T2)

Action planning was assessed by five items in each study. Items were derived from literature review [e.g., [50-52]] and expert consulting, and assessment was based on techniques used by van Osch and colleagues [34] and de Vries and colleagues [17]. Respondents were asked to what extent they planned to perform several actions or preparatory behaviors in order to reach the target behavior.

With regard to fruit consumption, specific actions that followed the item stem *'Do you have a plan to*...' pertained to 'buy (more) fruit?', 'eat fruit at a fixed time of day?', 'put a fruit basket on the table?', 'take fruit along with you when you go somewhere?', and 'replace unhealthy snacks by fruit?' (Cronbach's α = 0.75).

For snack consumption, action planning used the same item stem *'Do you have a plan to...'*, and specific actions pertained to 'buy less snacks?', 'buy healthy alternatives for snacks?', 'refrain from eating snacks at a fixed time of day?', 'substitute snacks by healthy alternatives?', and 'take healthy alternatives along with you when you go somewhere?' (Cronbach's α = 0.92). Answering options for all items ranged from 'I definitely do not' (1) to 'I definitely do' (7).

#### Fruit consumption (T1, T3)

The measurement of fruit consumption was based on a validated questionnaire [53] and comprised two items, referring to a) the amount of days in a week the respondent usually eats fruit (0 to 7), and b) the amount of fruit the respondent averagely consumes on each of these days. Multiplying the responses to these two questions gives a proper overview of the amount of fruit consumed during a week (Spearman correlation coefficients with two 24-hour consumption recalls = 0.68 for men, 0.75 for women; correct tertile classification = 52%) [53].

#### Snack consumption (T1, T3)

The measurement of snack consumption was based on previous questionnaires [49,54,55] and consisted of five items, measuring the consumption of five types of high-caloric snacks: 1. fatty snacks (e.g. hamburgers, pizza), 2. salty snacks (e.g. nuts, potato chips), 3. sugary snacks (e.g. cake, cookies), 4. candy bars, and 5. savory snacks (e.g. dices of cheese, sausage). Respondents were asked to indicate how many times per week they consumed each of the forenamed types of snacks. Answering options ranged from 'Never or less than once a week' (1) to 'Every day' (8). The five scores were added to indicate the total amount of snacks consumed per week.

### Statistical analysis

Structural Equation Modeling with Mplus 4.1 (Muthen & Muthen, 1998-2006), using Maximum Likelihood (ML) estimation was used to test hypothesized associations between the various cognitive constructs. Background variables (age, sex, and educational level) and behavioral measures of fruit and snack consumption were observed variables. Self-efficacy, intention, and action planning were latent constructs, measured by their separate indicators, as defined in the description of the questionnaires. In the basic models, intention and self-efficacy were modeled as direct predictors of behavior, whereas self-efficacy also had an indirect influence through intention. In order to assess the contribution of action planning in the prediction of both behaviors (hypothesis 1), a constrained pathway between action planning and the outcome behavior was included in the basic models. In the extended models, this relationship was freed and estimated. All models were corrected for the background variables. Past behavior was later added to the model and was thought to be correlated with intention and self-efficacy, and directly predictive of current behavior.

The moderation hypothesis (hypothesis 2) was tested using the Maximum Likelihood with robust standard errors and chi-square (MLR) because of the expected non-normality of the moderation model, as induced by the inclusion of the *intention × action planning *interaction term. The moderation models were compared to a constrained moderation model in which the interaction term was constrained to zero. The Satorra-Bentler scaled chi-square difference test was used to assess the statistical significance of the interaction term [56].

In order to test whether the predictive value of action planning was equally powerful for both behaviors (hypothesis 3), independent-samples comparison of correlation coefficients was performed [57].

Model fit was assessed using the Comparative Fit Index (CFI), the Tucker-Lewis-Index (TLI), and the Root-Mean-Square Error of Approximation (RMSEA). For a satisfactory model fit, the CFI and the TLI should be high (> 0.90), whereas the RMSEA should be low (preferably < 0.08) [58].

## Results

### Description of samples

Somewhat more than half of the respondents in the fruit consumption study were female (53.3%). The mean age of this sample was 47.8 years (SD = 16.0) and most respondents had a medium level of education (42.5%). Approximately one quarter of the respondents was of low educational level (26.3%) and 31.2% had a high educational level.

In the snack consumption study, 48.9% of respondents were female and the mean age was 49.5 (SD = 15.4). Again, most respondents had a medium level of education (37.6%), whereas 31.3% had a low level of education and 31.1% was highly educated. The mean intention towards eating sufficient amounts of fruit (Mean = 5.20; SD = 1.41) was substantially higher than the intention to restrict the consumption of high-caloric snacks (Mean = 3.18; SD = 1.62).

Chi-square difference tests and independent-samples t-tests did not indicate any demographic differences between the two study samples.

### Attrition analyses

Logistic regression analyses demonstrated that in the snack consumption study, drop-outs (N = 143) were somewhat lower educated than respondents who completed all three measurements (N = 442) (OR = 0.67, 95% CI = 0.98 - 1.02, *p *= 0.03). No differences were found between drop-outs and completers with regard to age, sex, self-efficacy, intention and snack consumption at baseline, and action planning at the first follow-up. No indications of selective attrition were found in the fruit consumption study.

### Measurement models

Bivariate correlations between cognitions and outcome behaviors are depicted in Table 1. Self-efficacy tended to correlate most strongly with fruit and snack consumption, whereas action planning correlated most strongly with intention.

**Table 1:** Pearson correlations between cognitions, past behavior and current outcome behaviors^a, b^

	**1**	**2**	**3**	**4**	**5**
1. Self-efficacy	-	0.58	0.40	0.63	0.57
2. Intention	0.38	-	0.48	0.42	0.36
3. Action planning	0.17	0.57	-	0.31	0.33
4. Past fruit/snack consumption	-0.42	-0.31	-0.18	-	0.76
5. Fruit/snack consumption	-0.36	-0.29	-0.22	0.60	-

^a ^All correlations between variables in the fruit consumption study are depicted above the diagonal; correlations between variables in the snack consumption study are depicted below the diagonal

^b ^All correlations are significant at the 0.001 level (two-tailed)

Confirmatory factor analyses were performed to test the measurement models with regard to both outcome behaviors. Both models included 11 items, measuring the three latent variables (self-efficacy, intention, and action planning). All factor loadings in both models were significant with values between 0.48 and 0.98 for the fruit consumption model and between 0.42 and 0.96 for the snack consumption model. The fit of both measurement models was satisfactory (fruit consumption: CFI = 0.98, TLI = 0.97, RMSEA = 0.06; snack consumption: CFI = 0.95, TLI = 0.93, RMSEA = 0.09).

### Model results: Fruit consumption

#### Basic model

The basic model with regard to fruit consumption fitted the data well (CFI = 0.95, TLI = 0.94, RMSEA = 0.06). Intention (β = 0.13; B = 0.62; *p *< 0.01) and self-efficacy (β = 0.47; B = 2.02; *p *< 0.001) were both significant predictors of fruit consumption, with self-efficacy as the strongest predictor. Age was the only significant demographic predictor of fruit consumption (β = 0.15; B = 0.06; *p *< 0.001). Together, they explained 36.2% of the variance in fruit consumption at T3, eight weeks after baseline.

#### Predictive value and mediating influence of action planning (hypothesis 1 and 2)

To assess the predictive value of action planning with regard to fruit consumption, action planning was modeled as a mediating variable between intention and behavior and between self-efficacy and behavior. The extended model fitted the data well (CFI = 0.96, TLI = 0.95, RMSEA = 0.06). Action planning significantly predicted fruit consumption (β = 0.20; B = 1.34; *p *< 0.001). Self-efficacy retained its behavioral impact (β = 0.42; B = 1.80; *p *< 0.001), whereas the influence of intention on fruit consumption was rendered non-significant (β = 0.05; B = 0.24; *p *> 0.10). This latter result indicates that action planning fully mediated the relationship between intention and behavior. Action planning itself was positively predicted by both intention (β = 0.42; B = 0.31; *p *< 0.001) and self-efficacy (β = 0.20; B = 0.13; *p *< 0.001). The extended model accounted for 39.3% of the variance in fruit consumption.

To test whether the inclusion of action planning made a significant contribution to the prediction of fruit consumption, a log-likelihood difference chi-square test was performed. This test resulted in a χ^2^-value of 12.32 (df = 1; *p *< 0.001), which indicates that adding action planning to the model significantly improved the prediction of fruit consumption.

**Figure 1:**
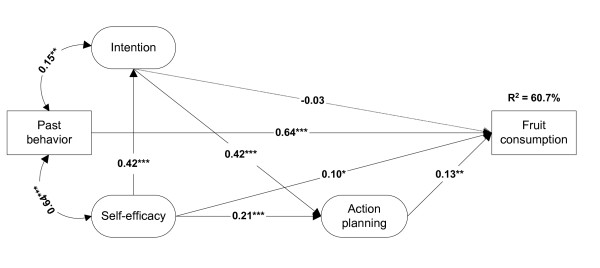
**Structural equation model with standardized regression coefficients assessing the predictive value of action planning with regard to fruit consumption**. *p < 0.05 **p < 0.01 ***p < 0.001

When past behavior was added to the model, action planning (β = 0.13; B = 0.87; *p *< 0.01) and self-efficacy (β = 0.10; B = 0.43; *p *< 0.05) remained significant (see Figure 1). Intention did not significantly predict behavior (β = -0.03; B = -0.12; *p *> 0.10). Past behavior was the most powerful predictor of fruit consumption (β = 0.64; B = 0.60; *p *< 0.001) and increased the explained variance of the model to 60.7%.

#### Moderating effect of action planning (hypothesis 2)

In order to estimate the potential moderating effect of action planning in the intention - behavior relationship, an *intention × action planning *interaction effect was added to the extended model (without past behavior). All other pathways were left unchanged. The interaction effect between action planning and intention was significant (t = 2.15; *p *< 0.05), indicating that action planning is more beneficial when intentions are high. We tested whether the inclusion of this interaction effect would result in a better model for explaining the role of action planning in the volitional phase. The Satorra-Bentler scaled chi-square difference test [56] was used to compare the extended model to the extended model with added interaction effect. In this test, the usual normal-theory chi-square statistic is divided by a scaling correction to better approximate chi-square under non-normality, as is the case when estimating an interaction effect. Calculation of the corrected difference in -2 log likelihood (Δ -2 LL = -5.04; df = 1; *p *< 0.01) indicated that including the action planning × intention interaction effect significantly improved model fit.

The moderating effect of action planning was also tested in the presence of past behavior. However, when past behavior was added to the model, the action planning × intention interaction effect was no longer significant (t = 0.80; *p *> 0.10).

### Model results: Snack consumption

#### Basic model

The basic model with regard to snack consumption fitted the data well (CFI = 0.94, TLI = 0.93, RMSEA = 0.07). Intention (β = -0.19; B = -0.44; *p *< 0.001) and self-efficacy (β = -0.31; B = -2.11; *p *< 0.001) were both significant predictors of snack consumption, with self-efficacy as the strongest predictor. The explained variance of snack consumption (16.8%) was substantially lower than that of fruit consumption.

#### Predictive value and mediating influence of action planning (hypothesis 1 and 2)

To assess the predictive value of planning with regard to snack consumption, action planning was modeled as a mediating variable between intention and behavior and between self-efficacy and behavior. This extended model fitted the data rather well (CFI = 0.95, TLI = 0.93, RMSEA = 0.07). Action planning was found to be a marginally significant negative predictor of snack consumption (β = -0.10; B = -0.27; *p *= 0.06), suggesting that higher scores on plans to restrict snack consumption lead to lower snack consumption. Self-efficacy (β = -0.31; B = -2.120; *p *< 0.001) retained its influence on behavior. Although intention also remained significant (β = -0.13; B = -0.30; *p *< 0.05), its predictive value was reduced as a result of the inclusion of action planning, indicating partial mediation of action planning in the intention - behavior relationship. Furthermore, intention was a strong predictor of action planning (β = 0.57; B = 0.51; *p *< 0.001), whereas the impact of self-efficacy on action planning was practically absent (β = -0.01; B = -0.03; *p *> 0.10). Together the behavioral determinants explained 17.5% of the variance in snack consumption.

To test whether the inclusion of action planning made a significant contribution to the prediction of snack consumption, a log-likelihood difference chi-square test was performed. This test resulted in a χ^2^-value of 3.51 (df = 1; *p *= 0.06), indicating that adding action planning to the model resulted in a marginally significant improvement of the prediction of snack consumption.

**Figure 2:**
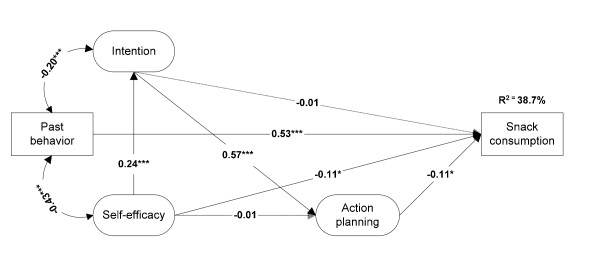
**Structural equation model with standardized regression coefficients assessing the predictive value of action planning with regard to snack consumption**. *p < 0.05 **p < 0.01 ***p < 0.001

When past behavior was added to the basic model, intention no longer significantly predicted behavior (β = -0.01; B = -0.03; *p *> 0.10; see Figure 2). Although past behavior was the most powerful predictor of snack consumption (β = 0.53; B = 0.48; *p *< 0.001) and increased the explained variance of the model to 38.7%, both action planning (β = -0.11; B = -0.28; *p *< 0.05) and self-efficacy (β = -0.11; B = -0.73; *p *< 0.05) were found to be significant predictors of behavior.

#### Moderating effect of action planning (hypothesis 2)

An *intention × action planning *interaction effect was included in the extended model to test for a potential moderating effect of action planning in the relationship between intention and snack consumption. Past behavior was initially excluded from the analyses; all other pathways were left unchanged.

A small trend towards significance was found for the interaction effect (t = -1.56; *p *= 0.12), which tentatively indicates that action planning may moderate the intention - behavior relationship. The Satorra-Bentler scaled chi-square difference test, however, indicated that the corrected difference in -2 log likelihood was non-significant (Δ -2 LL = 2.32; df = 1; *p *> 0.10) indicated that including the interaction effect did not improve model fit.

The moderating effect of action planning was also tested in the presence of past behavior. The action planning × intention interaction effect was not significant in this model (t = -0.49; *p *> 0.10).

#### Comparison of predictive value of action planning (hypothesis 3)

In order to test whether the predictive value of action planning differed with regard to the two behaviors, independent-samples comparison of correlation coefficients was performed [57]. This test resulted in a *z*-value of 1.50, indicating that the predictive value of action planning did not significantly differ between the two behaviors (*p *> 0.10).

## Discussion

The most important finding of the present study is that action planning significantly predicted health protective behavior (i.e. fruit consumption) as well as the restriction of health risk behavior (i.e., high-caloric snack consumption). Our results showed a better model fit when action plans were added to the model with only attitudes, social influences, self-efficacy and intentions, indicating that the prediction of both types of behavior significantly benefited from the incorporation of action planning, thereby conforming our first hypothesis. When viewed in the light of the literature on other health behaviors, such as physical activity [20-22,31], sun protection behavior [34,59], and (vitamin) pill intake [60,61], our findings with regard to fruit consumption support the notion that action planning may be an important strategy to promote health protective behaviors and suggest that current social-cognitive models on health protective behavior should be extended by incorporating volitional cognitions that facilitate the transition from motivation to behavior. Whereas most previous observational studies that found a behavioral influence of action planning failed to incorporate a measure of past behavior in the analyses, the present study accounted for the influence of past behavior in the extended analyses. Even after the inclusion of past behavior, which is generally the most powerful predictor of future behavior, action planning remained significant, which demonstrates that action planning significantly predicted behavior change. These findings corroborate results from several intervention studies, in which the formation of action plans has been shown to increase the performance of health behaviors [e.g., [20,25]]. The interplay between action planning and past behavior was outside the scope of the present study. Thorough examination of this relationship would, however, be an interesting direction for future research, as this may yield important information on theoretical modeling and practical application of planning strategies in individuals with high and low levels of past behavior.

Our findings with regard to snack consumption verify these suggestions and broaden their scope to include both health protective as well as health risk behaviors. The present study is the first to explicitly compare the predictive value of planning in both types of behaviors and found that the predictive value of action planning was equally powerful in the promotion of fruit consumption and the restriction of snack consumption. These findings confirm our third hypothesis and indicate that one and the same type of planning can be applied in both types of health behaviors.

Other important findings pertain to the established mediating and moderating effects of action planning (hypothesis 2). The longitudinal correlational design of the present study allowed us to examine the nature of the influence that action planning exerts in the intention - behavior relationship. Our findings of full mediation in the fruit consumption study and partial mediation in the snack consumption study confirm our hypothesis and correspond to results of previous studies, in which both full [e.g., [32,62,63]] and partial [e.g., [30,33]] mediation have been found in various behaviors. The difference in mediating effects may pertain to the strength of the underlying intentions. Wiedemann and colleagues [64] have demonstrated that the strength of the mediated effect of action planning increases along with levels of intentions. The relatively low intention with regard to restricted snack consumption, as compared to fruit consumption, may therefore have precluded full mediation of the intention - behavior relationship by action planning.

The results with regard to potential moderating effects of action planning partially confirm our second hypothesis. A positive moderating effect of action planning was demonstrated in the fruit consumption study, thereby replicating previous reports of moderation of the intention - behavior relationship [e.g., [30,33]]. However, only a small trend with regard to the moderation effect was found in the snack consumption study. The insignificance of this effect may, again, be explained by relatively low motivation scores; the overall intention towards restricted snack consumption was substantially lower than the intention to eat sufficient amounts of fruit, which may have precluded the appearance of moderating effects of action planning in the snack consumption study. Besides the proposition to incorporate action planning in existing, traditional social-cognitive models, these findings provide suggestions on how and where to integrate the concept; action planning can tentatively be considered as a mediator as well as moderator in the intention - behavior relationship. It should, however, be mentioned that the present consideration of action planning as concurrent mediator and moderator, is at odds with the conceptualization of moderators as being unaffected by the status of a predictor variable [e.g., [65,66]; but see [67,68]]. In the present study, action planning was measured at T2 in order to adequately investigate its mediating influence. Although this measure may be tentatively considered as a proxy for a baseline measure of action planning, application of the latter would have resulted in a stricter conceptualization and testing of the moderation effect. This limitation should be taken into account when interpreting the current findings and future studies would do well to incorporate longitudinal measurements of mediating and moderating variables.

Furthermore, whereas the four previous studies used a similar type of action planning [i.e. implementation intentions; [24,26,35,36]], the current study used a different approach. Instead of focusing on when, where, and how a goal-directed response will be implemented (i.e. eating fruit, not eating snacks), the formation of specific preparatory plans was emphasized. Although the former type of planning, i.e. implemental planning, has been subject of substantial research efforts to decrease to intention - behavior gap, the latter planning mode, i.e. preparatory planning, has also been shown to reliably predict a variety of health behaviors [e.g., [17,34,39]]. Moreover, one of our previous studies compared the behavioral influence of both types of behaviors and found that preparatory planning outperformed implemental planning in the prediction of fruit consumption [69]. Further, preferably experimental, research is, however, recommended to substantiate the present findings and optimize planning concepts and interventions for both health protective and health risk behaviors. In doing so, the application of coping planning as a protocol for restriction of health risk behavior may be reckoned with. Coping planning is a barrier-focused strategy that pertains to the identification of risk situations and the specification of suitable coping responses [70]. As this strategy has been shown to reliably predict performance of health behavior in the face of barriers [20,70-72] and has been successfully applied to the restriction of health risk behavior, such as smoking [73] and binge-drinking [74,75], comparison of the benefits of this and other types of planning may yield vital knowledge for the optimization of planning interventions.

Limitations of the present study need to be acknowledged. First, the lack of validity statistics with regard to the behavioral assessment of snack consumption should be mentioned and calls for caution in the interpretation of the results regarding this measure. Items used in the present study were part of a food frequency questionnaire to estimate total and saturated fat intake [54,55] and although this questionnaire has been previously validated, there are currently no specific validity statistics available for the selection of items used to measure snack consumption.

Second, a relatively low explained variance of snacking behavior was found, indicating that other motivational, volitional, and/or environmental factors need to be taken into account for the prediction of snack consumption. The low explained variance is, however, not uncommon, as dietary behavior is generally not well-predicted with explained variances of 30% and higher being exceptions rather than the rule [76]. Furthermore, although ultimately this study aims at optimizing the prediction of fruit and snack consumption, the primary purpose was to investigate the influence of action planning in the intention - behavior relationship. We therefore only took three other direct predictors of the behaviors into account (past behavior, intention and self-efficacy), whereas most previous studies included many more determinants, often resulting in higher explained variances [e.g., [49,54,77]]. Third, data were collected from a random sample of adults that were all members of an existing internet research panel. As these respondents voluntarily participate in surveys and receive incentives for their participation, the degree to which the findings generalize to the Dutch population at large may be limited. However, the demographic characteristics of the participants in both study samples corresponded rather well to demographic distributions within the Dutch adult population [5], rendering substantial reduction of the external validity of our results unlikely. Furthermore, attrition was found to be somewhat selective in the snack consumption sample as lower educated participants were more likely to drop out. This attrition bias may limit internal and external validity of the study. However, general attrition rates were equal in both study samples and the influence of educational level as a covariate was not significant. It is therefore unlikely that the main results of this study have been compromised as a result of attrition.

## Conclusion

Although replication of the findings in preferably experimental settings is required for different behaviors as well as different types of action planning, the present study indicates that action planning may benefit both the actual performance and initiation of healthy behavior and the restriction and suppression of unhealthy behavior. Future interventions in dietary modification may turn these findings to advantage by incorporating one common planning protocol to increase the likelihood that good intentions are translated into healthy dietary behavior.

## Competing interests

The authors declare that they have no competing interests.

## Authors' contributions

LvO coordinated the conception and design of the study and the acquisition of the data. Furthermore, LvO analyzed and interpreted the data and drafted the manuscript. MB participated in the data analysis and data interpretation and the drafting of the manuscript. AR and LL participated in the design of the study and the acquisition of the data and critically revised the manuscript. MC substantially participated in the analysis and interpretation of the data and helped to draft the manuscript. HdV was involved in the design of the study and the acquisition of the data and critically revised the manuscript. All authors read and approved the final manuscript.
